# Research advances in the microbiota-gut-brain axis and Parkinson's disease (Review)

**DOI:** 10.3892/mmr.2026.14016

**Published:** 2026-09-10

**Authors:** Yangping Meng, Qian Liu, Hao Zou, Xingyu Zhu, Xinxin Mi, Sitong Li, Yutian Xia, Yucheng Ma, Qianyu Yang, Jiaxin Peng, Junrui Li, Xingliang Liu, Junli Chen

**Affiliations:** 1 Department of Pathophysiology, West China School of Basic Medical Sciences and Forensic Medicine, Sichuan University, Chengdu, Sichuan 610041, P.R. China; 2 Department of Neurology, The First Affiliated Hospital of Hebei North University, Zhangjiakou, Hebei 075061, P.R. China

**Keywords:** Parkinson's disease, microbiota-gut-brain axis, gut microbiota, therapy, α-synuclein

## Abstract

Parkinson's disease (PD) is a common neurodegenerative disease with diverse pathogenic mechanisms. The microbiota-gut-brain axis is closely related to the development of PD, in which non-motor symptoms are considered to be the early manifestation and exacerbation of the disease. Nevertheless, reliable diagnostic criteria or biomarkers for PD have not been fully developed. The gut microbiota as a key transmitter of the gut-brain axis, can affect disease progression through a variety of pathways, and may be a potential therapeutic target for PD. Therefore, new strategies have been developed with the aim to prevent and control PD by modulating the intestinal microflora, using tools such as antibiotics, probiotics, prebiotics, dietary interventions, fecal microbiota transplantation and vagus nerve stimulation; however, these interventions also face certain issues and challenges. The present article reviewed the latest research on PD through the microbiome-gut-brain axis, offering new angles for understanding and treating the condition.

## 1. Introduction

Parkinson's disease (PD) is a progressive neurodegenerative disorder that affects a marked number of patients, with data indicating that the prevalence of PD among individuals aged 65 years and older is >2% (1). China harbors a particularly high proportion of patients with PD, with projections indicating that by 2030 the country will account for two-thirds of the global PD population (2). A global epidemiological analysis revealed that, since the start of the 21st century, the annual prevalence growth rate has been notably higher than that of the preceding century (an annualized growth of 16.32% from 2004 to 2023), with prevalence reaching a peak between 2010 and 2023, at 3.81/1,000 individuals (3). PD severely diminishes the quality of life for patients and imposes a substantial economic burden on healthcare and public health systems. Finding effective, safe and feasible treatment options represents an urgent medical challenge needs to be addressed.

PD is typically diagnosed using the UK Brain Bank criteria, which demonstrates a sensitivity of >80%. Alternatively, the 2015 Movement Disorders Society criteria introduced the concept of preclinical stage and achieved an accuracy rate of 92.6%. Prospective studies indicate that these criteria are >98% specific in all cases (4–6). At present, it is generally considered that standardized neuropathological testing markers (such as bodily fluid biomarkers) are not yet clinically applicable. Early diagnosis of PD remains a major clinical challenge for two key reasons. First, mainstream clinical diagnostic criteria exhibit poor reliability and consistency owing to interference from multiple external confounding factors, resulting in high rates of misdiagnosis and missed diagnosis (7). Second, no universal gold standard has been established for disease-specific biomarkers, and strict control of biochemical testing conditions remains challenging. Accordingly, developing high-sensitivity multi-level biomolecular detection approaches for PD diagnosis may effectively lower misdiagnosis rates and minimize the bias caused by the subjective judgments of researchers relying on early clinical symptoms. Technological advances and the emergence of multi-source data have accelerated the development of multimodal fusion, a sophisticated approach that integrates structural and functional information to enable multidimensional comprehensive characterization of PD. This approach supports the formulation of more accurate personalized diagnostic and therapeutic regimens. Recently, Wang *et al* (8) proposed the Spatial-temporal Dual-pathway Network with Multi-scale Feature Fusion method. This model yielded a diagnostic accuracy of 0.926 on public datasets and 0.858 on private datasets, confirming its notable performance for PD diagnosis improvement (9).

**Figure 1.**
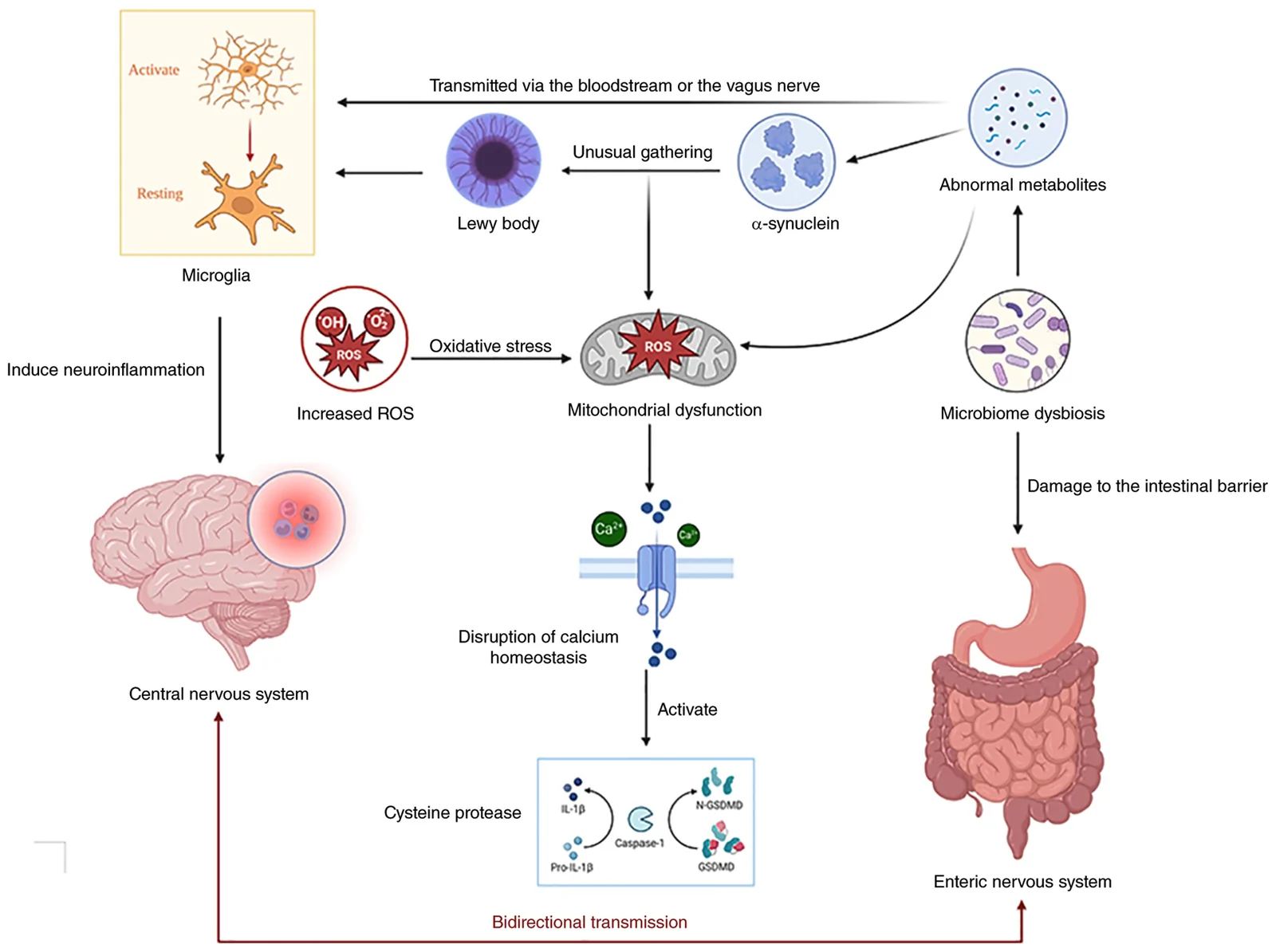
Pathogenic mechanisms of Parkinson's Disease. The flowchart illustrates that PD is associated with multiple interacting mechanisms, involving multiple levels such as abnormal protein expression and aggregation, neuroinflammation, oxidative stress, gut dysbiosis and mitochondrial dysfunction. ROS, reactive oxygen species.

A review of prior clinical cases indicates that the hallmark clinical manifestations of PD center on motor function impairments, including bradykinesia, resting tremor, gait and balance disturbances (10). Olfactory dysfunction is recognized as an early biomarker of PD and is among the earliest non-motor symptoms of the disease (11). During the early disease stage, patients frequently exhibit a spectrum of non-motor symptoms, such as constipation, nausea, urinary dysfunction, sleep disturbances, depression, anxiety and hyposmia (12). Furthermore, patients in both motor-predominant and neuropsychiatric symptom-predominant subtypes displayed more notable motor deficits, anxiety and depressive symptoms at baseline. Such phenotypic characteristics may signal autonomic neuropathy-mediated non-motor symptoms (13). The Movement Disorder Society Task Force attributes the onset of the ‘prodromal phase’ to alterations in the nondopaminergic nervous system, which constitutes a distinct clinical stage preceding the persistent injury and loss of dopaminergic neurons in the substantia nigra pars compacta (SNPC) (6). Via single-molecule optical imaging, prominent disparities in the size and abundance of α-synuclein (α-Syn) aggregates were detected in tissue samples collected from patients with PD vs. healthy control participants (14). This pathological progression evidence is vital for clarifying the underlying pathogenesis of PD. Given that PD pathogenesis involves multi-level interactive mechanisms (Fig. 1), the existing experimental data remain insufficient to fully decipher its fundamental etiology.

By the time patients develop motor symptoms, 50–70% of dopaminergic neurons within the SNPC already present with dysfunction or complete loss of activity (15–17). Kim *et al* (18) demonstrated that intestinal injection of pathological α-syn in mice triggers its retrograde transport along the vagus nerve to the brainstem solitary tract nucleus, ultimately reaching the SNPC. These findings validate the gut-to-brain propagating pathway of α-Syn pathology and substantiate the pathogenic hypothesis of idiopathic PD. Accordingly, timely identification, diagnosis and intervention during the prodromal phase marked by non-motor symptoms may delay or even halt PD disease progression.

The emergence of these non-motor manifestations is associated with alterations in the composition of gastrointestinal (GI) microbiota and their derived metabolites in patients with PD. Tremlett *et al* (19) theorized that GI dysfunction plays a pivotal role in the onset and progression of neurodegenerative diseases and is tightly linked to disease onset risk, recurrence and progression. A growing body of preclinical studies demonstrates that the gut microbiota (GM) participates in gut-brain signaling via multiple mechanisms, among which endocrine and neurosecretory pathways exert dominant effects. In such gut-brain axis crosstalk, the central nervous system (CNS) can further modulate gut microbial composition and activities through the autonomic nervous system (ANS). Researchers have proposed the microbiota-gut-brain axis (MGBA) as a theoretical extension based on the aforementioned biological mechanisms (20). This framework highlights the bidirectional communication network connecting the enteric nervous system (ENS) within the GI tract and CNS. Dysbiosis of GM and its metabolites facilitate PD pathogenesis by modulating neuroinflammation, intestinal barrier integrity and neurotransmitter functions along this signaling pathway.

Novel PD therapeutic strategies targeting the gut-brain axis have emerged and undergone continuous optimization and innovation, with promising application prospects (21,22). The present review aimed to summarize MGBA-related PD mechanisms, lays a solid theoretical basis and offers critical insights for drug development, and aims to systematically elaborate the MGBA-PD association to facilitate the discovery of more efficacious PD treatment regimens.

## 2. MGBA

The human GI is a complex and dynamic ecosystem that houses >100 trillion microorganisms, including bacteria, fungi, protozoa and viruses (23). The biological importance and research potential of these microbial communities have been well established and validated from multiple perspectives (24–26). Liu *et al* (27) identified that shifts in GM composition regulate neurotransmitter synthesis and secretion. For instance, these alterations trigger protein aggregation and further induce neurological dysfunction. Additionally, in neurodegenerative diseases including PD, GM modulates gut-brain crosstalk via multiple pathways, including immune pathways, the vagus nerve and the circulatory system (28). This finding indirectly supports the multi-pathogenic mechanisms of PD. Hence, GM exerts an indispensable effect on maintaining basic physiological functions and bodily health.

Nevertheless, diverse factors affect the composition, abundance and physiological functions of intestinal bacterial genera. These factors include age, diet, genetic background, physiological status, lifestyle and environmental exposure. GI function is not controlled by a single independent system. Instead, GI activities are coordinately modulated by three core systems: i) CNS; ii) ANS; and iii) ENS. The crosstalk among these three systems is critical for regulating and sustaining normal GI functions (29).

The ENS is a complex semi-autonomous neural network widely distributed along the digestive tract, consisting of the submucosal and myenteric plexuses in the esophagus, stomach, small intestine and colon (30). The ENS can function independently of the CNS and comprises >26 subtypes of intestinal neuron, including motor neurons, interneurons and primary afferent neurons; these neurons mediate the well-documented gut-gut reflex (31). Additionally, the communication process is facilitated by the ANS and the hypothalamic-pituitary-adrenal axis, thereby linking the peripheral gut to the CNS (32).

As a core physiological network, the gut-brain axis connects behavioral, cognitive and emotional activities with GI functions via the interplay of neurotransmitters, hormones and neural signals (33). The vagus nerve, a major mixed cranial nerve, serves as the primary communication conduit between the gut and the brain. It mediates direct gut-brain crosstalk and builds an elaborate regulatory network to facilitate signal transmission and functional coordination (34). The GM modulates host physiological functions via this network through direct and indirect signaling pathways, including chemical mediators, immune responses and neural circuits (35). Abnormal alterations or ecological dysregulation of GM composition have been demonstrated to induce a range of conditions, including PD, Alzheimer's disease, anxiety, depression and autism. Alterations on these signaling pathways, and fluctuations in GM are concomitant with the progression of neurodegenerative diseases (36,37). For instance, GM-derived tryptophanase and aminotransferase act synergistically to metabolize tryptophan into indole and its derivatives, including indole-3-acetaldehyde, indole-3-acetic acid and indole-3-lactic acid. Bacterial genera such as *Escherichia coli, Bacteroides spp., Bifidobacterium* and *Clostridium* play notable roles in this conversion process (38). These substances can be absorbed into the bloodstream via the aryl hydrocarbon receptor (AHR) and subsequently mediate various cellular physiological activities. Among these, tryptophan metabolites have been shown to protect intestinal barrier function via AHR. Therefore, modulating indole levels may improve the prognosis of CNS disorders and restore functional dysregulation of the MGBA (39,40).

## 3. MGBA and PD

### Microbial

To further elucidate interactions among microbial communities, researchers have adopted computational approaches to assess their biosynthetic potential. These efforts help simulate molecular crosstalk within and across microbial populations (41). Multimodal omics approaches, encompassing genomics, transcriptomics and epigenomics, facilitate the acquisition and analysis of disease-specific data, thereby unraveling intricate biological regulatory networks (42). Using multimodal omics approaches, Hensen and Thiele (43) profiled PD-related metabolites and reconstructed whole-body metabolic models using gut metagenomic datasets. The aforementioned study screened six blood metabolites with the most notable discrepancies compared with the control group and identified key microbial species that mediate such metabolic alterations (Table I). This technology provides a novel and effective computational tool for investigating the correlation between GM and PD-related metabolic markers, offering theoretical insights for the development of novel therapies and interventions targeting the gut-brain axis.

The contribution of these GM to metabolic processes is likely to be influenced by shifts in their relative abundance (44). Accumulating clinical and pathological evidence reveals that patients with PD commonly present with altered GM composition and metabolic activity, which serve as critical contributors to GI dysfunction (45). Qian *et al* (46) comprehensively analyzed the composition of the GM in Chinese patients with PD. The results demonstrated that patients with PD had markedly higher GM richness and diversity than healthy controls Notable disparities were detected in five bacterial genera, including *Klebsiella*. Moreover, most identified genera were positively associated with disease duration, while *Bacteroides* and *Corynebacterium* were negatively associated with an equivalent dose of levodopa. Collectively, these findings indicate that gut metabolic alterations in PD are induced not by a single microbial strain, but rather by the synergistic effects of multiple microbial species.

A meta-analysis integrating large-scale machine learning tools and metagenomic data on PD-related microbiome characteristics reveals that alterations in specific microbial pathways trigger intestinal disorders and facilitate the transmission of pathogenic molecules via the gut-brain axis (47). Intestinal permeability changes mainly stem from GM dysbiosis or impaired intestinal barrier integrity. For instance, intestinal bacteria such as *Clostridium scindens* are capable of converting primary bile acids into secondary bile acids, namely deoxycholic acid and lithocholic acid, which can disrupt tight junction proteins (48). Microbial metabolites and endotoxins represented by lipopolysaccharide (LPS) can enter the bloodstream and trigger the release of inflammatory mediators within the intestinal tract. This process elevates the risk of PD in individuals with pre-existing inflammatory disorders such as inflammatory bowel disease and also increases susceptibility to inflammatory diseases of the CNS (49). Furthermore, metabolic byproducts derived from pathogenic bacteria can enter the brain directly via the circulation and induce neuroinflammation. Specifically, these metabolites activate resident immune cells in the CNS, particularly microglia, disrupting blood-brain barrier (BBB) integrity (36). In addition, damage to the nervous system itself may consequently induce systemic inflammation, resulting in fluctuations in the microecological balance of the gut (50). It is imperative to acknowledge the importance of systemic inflammation, triggered by ecological imbalance, in exacerbating oxidative stress within the CNS. Given that microbial metabolites exert regulatory effects on astrocyte function, Zhang *et al* (51) proposed the microbiota-astrocyte axis to elucidate its regulatory role in cognitive impairment during the progression of neurodegenerative disorders. This novel axis is expected to provide a promising target for future PD therapeutic interventions.

Short-chain fatty acids (SCFAs) are major metabolites generated by GM during dietary fiber fermentation. They are notably linked to host metabolism, immune modulation and intestinal epithelial integrity (52). GM dysbiosis reduces the abundance of SCFAs, represented by butyrate, thereby increasing BBB permeability and aggravating neuroinflammatory responses (53). Collectively, GM modulate PD progression by altering circulating microbial metabolites. SCFAs have several well-established potent neuroprotective properties, including exertion of anti-inflammatory effects, improvement of mitochondrial function, preservation of BBB integrity and regulation of neurotransmitter signaling (54). Accordingly, SCFA-based therapies offer great potential for treating PD and other neurodegenerative diseases.

### Intestinal barrier

The intestinal barrier serves as a core structure maintaining intestinal internal homeostasis, which can be divided into mechanical and biological barriers. The mechanical barrier mainly consists of the mucus layer and monolayer intestinal epithelial cells connected via tight junctions, whereas the biological barrier comprises GM and its dynamic interactions with the host (55). In combination, these two barriers modulate nutrient absorption and block pathogenic invasion, indicating a tight association between intact barrier function and host physical health (56). Metabolites derived from GM, including SCFAs, indole derivatives and bile acids, maintain intestinal barrier integrity by modulating tight junction protein expression, mucus secretion and epithelial repair (57). Furthermore, mucin, a major component of the mucus layer, is required for sustaining epithelial barrier function. As a key regulatory molecule, it interacts with microbes and host cells to dynamically modulate intestinal barrier permeability. Aberrant mucin expression initiates a cascade of pathological events, including impaired intestinal barrier function, GM dysbiosis and increased intestinal permeability, which collectively facilitate the occurrence of various GI disorders (58,59).

ENS plays a notable role in regulating the function of the intestinal epithelial barrier. The intestinal barrier functions as an integrated system rather than an isolated structure. Its surface is lined with an intricate network of intestinal neurons and glial cells, collectively forming a ‘neuro-epithelial barrier’ (60). This structure plays a role in isolating internal and external environments and maintaining intestinal homeostasis. For example, impaired intestinal barrier integrity and elevated intestinal permeability permit harmful metabolites (such as LPS), to cross the barrier and infiltrate the ENS. Upon ascending to the CNS, these toxins disrupt BBB homeostasis, compromise the physiological functions of astrocytes and microglia, trigger dopaminergic neuronal apoptosis and provoke neuroinflammation (61,62). This process is associated with the pathogenesis of neurodegenerative diseases (such as PD, Alzheimer's disease and multiple sclerosis) (63,64), indicating that intestinal barrier dysfunction and GM dysbiosis are key mechanisms driving the onset and progression of PD (65,66). Notably, indole exerts adverse impacts on the intestinal barrier under certain conditions (67). Emerging evidence indicates that beneficial indole metabolites preserve intestinal barrier integrity and the BBB, whereas toxic indole derivatives trigger α-Syn aggregation and neuronal loss. Dysregulation of these indole species promotes the pathogenesis of PD (68). This bidirectional dual effect highlights the necessity of maintaining a delicate intestinal chemical balance for disease prevention and improved prognosis.

### α-Syn

α-Syn is a presynaptic neuronal protein that exists in dynamic equilibrium between soluble and membrane-bound forms. Under certain pathological conditions, α-Syn undergoes misfolding, oligomerization and fibrillation, generating toxic species that propagate across brain regions. In addition, pathogenic α-Syn can spread to the CNS via the vagus nerve in a prion-like manner, exacerbating neurodegeneration in interconnected neural circuits (69–71). α-Syn is also a key constituent of Lewy bodies, which are cytoplasmic inclusions located in the substantia nigra and serve as an early pathological hallmark of PD. They trigger neuroinflammation and oxidative stress through microglial activation, thereby increasing the risk of PD onset (72,73). In PD mouse models, GM depletion mitigates motor dysfunction and abnormal α-Syn aggregation relative to conventional mice with intact GM (74). Accumulating evidence has indicated that GM-derived SCFAs can modulate α-Syn aggregation. Furthermore, SCFAs are involved in inflammatory and immune responses, and are capable of regulating disease progression as well as protein neurotoxicity (75). Consequently, restoring GI physiological homeostasis holds potential for suppressing PD pathological progression.

Pathological studies have revealed α-Syn aggregates in the myenteric plexus of all patients with PD. Numerous differentially expressed genes were found in the intestinal myenteric plexus of patients vs. healthy controls. Upregulated genes including LAMP1 and TUBB2A are chiefly enriched in pathways mediating neuroepithelial differentiation and axonal development (76). These findings suggest that α-Syn deposition may trigger active neural regeneration in the gut. Balsamo *et al* (77) demonstrated that intestinal α-syn aggregation is linked to dopamine biosynthesis in enteroendocrine cells. Following vagotomy, the brain burden of α-Syn aggregates is notably reduced. This study further validated that the vagus nerve serves as the critical anatomical pathway mediating the gut-to-brain propagation of α-Syn aggregates. In addition, the overexpression of Lewy neurons and Lewy bodies in the CNS is associated with BBB dysfunction (78); however, it is difficult to clarify the temporal sequence between BBB impairment and α-Syn aggregation. This can be attributed to inherent discrepancies between *in vivo* and postmortem detection approaches, as well as region-specific heterogeneity in BBB integrity across individual brains. Such inconsistencies may obscure subtle pathological alterations when whole-brain tissue is analyzed.

Höglinger *et al* (79) confirmed that detecting pathological α-Syn in patients with PD should serve as a key molecular basis for disease classification. Patients who are diagnosed as α-Syn positive should be categorized as S+, while all other cases should be classified as α-Syn negative (S-). This biological classification system facilitates the execution of discrete studies by researchers on patients with sporadic PD, hereditary PD, asymptomatic α-Syn-positive PD and other patient types. Consequently, this enables the development of targeted diagnostic and treatment plans.

## 4. Diagnostic and therapeutic strategies based on the MGBA

**Figure 2.**
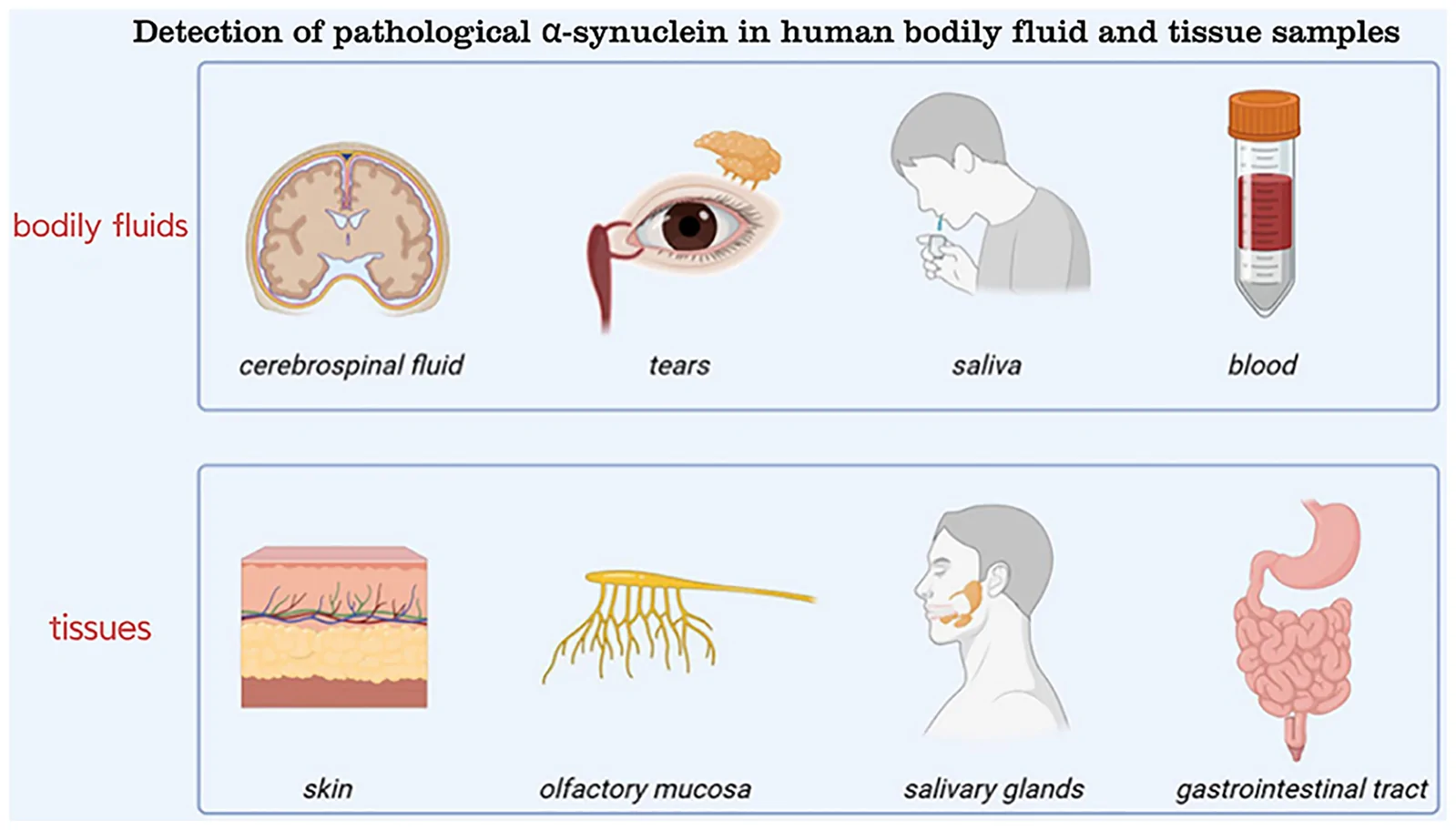
Detection of Pathological α-Syn in Human Bodily Fluid and Tissue Samples. The diagram enumerates common human samples for pathological α-Syn testing, covering four types of bodily fluids (cerebrospinal fluid, tears, saliva, blood) and four categories of tissues (skin, olfactory mucosa, salivary glands, gastrointestinal tract).

The staging and diagnosis of neurodegenerative diseases primarily rely on liquid biopsy, which requires biomarkers released into body fluids to exhibit high specificity and sensitivity (80,81). Currently, numerous methodologies have been utilized to detect pathological α-Syn in biological fluids and tissues (Fig. 2) (79). Over the years, techniques with enhanced sensitivity and specificity for detecting synuclein-related biomarkers in living patients have emerged. The Seed Amplification Assay (SAA) represents a robust diagnostic approach that exploits the prion-like properties of misfolded proteins for *in vitro* signal amplification. As one of the most transformative tools in α-Syn research, SAA has driven a paradigm shift in the biological diagnosis of PD (82–85). Among these, detection in skin and cerebrospinal fluid exhibits the highest sensitivity, at 0.92 and 0.90, respectively (86). Concurrently, monitoring changes in pro-inflammatory biomarkers (such as IL-1β, IL-6, IL-10, TNF-α and RANTES) in cerebrospinal fluid or blood, along with alterations in GM characteristics (87), plays a role in facilitating the early diagnosis of PD and enabling continuous monitoring of disease progression. However, due to factors such as inter-individual biological heterogeneity and the complexity of detection technologies, existing evidence does not support the use of the qualitative and quantitative results of these biomarkers as a reliable basis for distinguishing patient populations from healthy individuals (88).

High-sensitivity C-reactive protein and soluble TNF-receptors can also be utilized as potential peripheral biomarkers in instances where there is an absence of accurate and reliable biomarkers for early diagnosis based on GM associations (89). Sequencing and multimodal omics approaches are continually evolving. Liu *et al* (90) systematically summarized gut metagenomic profiles of neurodegenerative diseases by integrating bioinformatics analyses and wet-lab experiments. High-throughput sequencing further uncovered the complex bidirectional crosstalk between the CNS and ENS (90). Qian *et al* (91) identified 25 potential genetic biomarkers from macrogenomic species to distinguish PD from non-PD populations using bioinformatic analyses and quantitative polymerase chain reaction methods.

Sleep disorders rank among the most prevalent non-motor complications of PD, emerging across the entire disease spectrum, including in the prodromal phase prior to motor symptom onset, the active stage and the advanced stage. Clinical studies show that 80–90% of patients with PD are affected by such conditions, including rapid eye movement sleep behavior disorder (RBD), circadian clock dysfunction, obstructive sleep apnea, insomnia and nightmares (92–94). As neurodegenerative diseases advance, the incidence of these sleep disorders rises accordingly. Circadian clock dysfunction is ubiquitous in patients with PD, as they aggravate motor and non-motor symptoms and potentially drive disease progression (95). Studies have identified abnormal α-Syn pathway activity in patients with RBD, along with inflammation and mitochondrial dysfunction (96). Additionally, RBD affects disease severity and progression and is notably associated with impaired cognitive function and autonomic dysfunction, as well as cortical atrophy (97,98). A randomized controlled trial (RCT) showed a higher prevalence of α-Syn in patients with early-to mid-stage PD and RBD (84%) and in patients with RBD and undiagnosed PD (79%), compared with patients with PD without RBD (54%) (99). This phenomenon may stem from the transmission of α-Syn through the peripheral nervous system, which leads to the hypothesis that there is a correlation between RBD and changes in intestinal flora. At present, multiple effective symptomatic treatments have been established. Strategies including improving sleep quality, ensuring adequate sleep and maintaining regular sleep patterns help to preserve long-term brain health. Hence, patients with RBD will be a focus in neuroprotective treatment trials (100). In summary, current evidence validates RBD as a markedly specific prodromal biomarker for PD and underscores its central role in α-Syn-targeted therapeutic strategies (101).

The pipeline for developing new medical therapies and drugs for PD is well-established. Nevertheless, further progress is required in the testing of treatments that slow disease progression in later stages (102). Exercise plays a marked role in managing PD (103) and combining personalized rehabilitation approaches with routine medical treatment can lead to safer and more effective therapeutic outcomes (104). In a recent review, Festa *et al* (105) proposed therapeutic interventions targeting oligodendrocytes to restore their neurofunctional capacity. Meanwhile, de Bie *et al* (106) updated evidence-based medical recommendations for treating motor fluctuations in PD. This analysis included 102 studies on improving motor symptoms in patients with PD. The studies examined pharmacological treatments such as levodopa in sustained-release and immediate-release formulations, prucalopride in sustained-release and immediate-release formulations, and interventions such as continuous enteral levodopa infusion and bilateral globus pallidus deep brain stimulation (106). The strengths of this assessment lie in its adoption of new methodology, including the Cochrane risk-of-bias tool and the updated GRADE system. These approaches help clinicians and patients to select personalized treatment strategies and provide researchers with insights for developing novel interventions in the future.

A growing number of novel adjunctive therapeutic strategies with proven efficacy in modulating the microbiome are being explored in clinical research. The present review introduces several extensively studied approaches that have progressed, including pharmacological methods such as probiotics, prebiotics and synbiotics, and non-pharmacological methods such as dietary interventions, fecal microbiota transplantation (FMT) and vagus nerve stimulation (VNS) (107).

### Prebiotic interventions

Over the years, researchers have focused on the role of prebiotics in regulating intestinal flora. Prebiotics are dietary ingredients that are not digested or absorbed by the body, yet selectively stimulate the growth or activity of beneficial bacteria (such as *Lactobacillus* spp. and *Bifidobacterium* spp.) in the intestinal tract, thereby positively affecting host health (108). The predominant categories of prebiotics include fructans (encompassing inulin and oligofructans), oligogalactans, glucose-derived oligosaccharides (such as polydextrose) and pectic oligosaccharides (109). These compounds occur naturally in numerous food products, such as garlic, onion, chicory, asparagus, artichoke, tomato, wheat, barley and rye (110). These bacteria have been demonstrated to improve bowel habits, inhibit the growth of pathogens and improve intestinal barrier function (111). In a mouse model with α-Syn overexpression, a high-fiber prebiotic diet ameliorated motor deficits, attenuated α-Syn aggregation in the substantia nigra and regulated microglial function (112). In a murine model of chronic PD, five weeks of oral treatment with polymannuronic acid prebiotic plus the probiotic *Lacticaseibacillus rhamnosus* protected dopaminergic neurons from injury (113). These findings indicate that prebiotics exert moderate neuroprotective effects.

It is hypothesized that prebiotics are both safe and effective in treating constipation. In a RCT, 74 patients with PD were allocated to the following three groups: i) Standard diet; ii) resistant starch supplementation; and iii) high-fiber diet. Multi-omics analyses of fecal metagenomes and metabolites were conducted to evaluate short-term (13 weeks) and long-term (>13 weeks) effects. The results demonstrated that resistant starch supplementation raised the abundance of SCFA-producing *Faecalibacterium* and triggered favorable alterations in fecal metabolites (including butyrate, tryptophan metabolites and bile acid). Long-term intervention further remodeled the GM, regulated inflammation and ameliorated PD symptoms (114).

Perez-Pardo *et al* (115) reported that the motor symptoms of patients with PD notably improved following prebiotic intervention. Furthermore, prebiotic fiber can be administered as a dietary supplement to boost the production of SCFAs, including acetate, propionate and butyrate. This intervention replenishes SCFA-synthesizing bacteria in patients with PD and restores GM dysbiosis. Nevertheless, this strategy has limitations, as it only yields mild improvements in motor symptoms (116).

To conclude, preclinical and clinical investigations share consistent research trends yet vary in the strength of evidence. Prebiotic supplementation consistently improves motor performance, alleviates neuroinflammation and preserves dopaminergic neurons in animal models, with systematic reviews reporting positive results in >96% of these studies (117). By contrast, evidence from human trials is limited, and follow-up periods are insufficient to assess long-term safety and sustained efficacy. Furthermore, while animal studies can assess multiple ‘gold standard’ neuroprotective endpoints, RCTs can only make indirect inferences based on the Movement Disorder Society-Unified Parkinson's Disease Rating Scale (MDS-UPDRS), fecal metabolites and quality-of-life questionnaires. These findings highlight the need in this field for larger-sample RCTs, standardized intervention protocols and longer-term follow-up studies to confirm efficacy, elucidate mechanisms and bridge the translation gap. Given the considerable therapeutic potential of prebiotics in GI disorders, future studies should focus on identifying the specific microorganisms that utilize distinct prebiotics as nutritional substrates and the mechanisms by which these supply pathways are influenced by factors such as microbial interactions, sex, age, dietary habits and other variations.

### Probiotic interventions

Probiotics are live microorganisms that exert health-promoting effects in the host, forming the foundation for the research and development of live biotherapeutic products and prebiotics. Unlike prebiotics, probiotics do not merely supply nutrients, they introduce viable microorganisms directly into the host (118). Probiotics can colonize the human gut and other bodily sites, and secrete a variety of bioactive substances, including GABA, serotonin, tryptophan, dopamine and acetylcholine. These metabolites help maintain local microbiota homeostasis and suppress the proliferation of pathogenic bacteria (119). *Lactobacillus* and *Bifidobacterium*, the most widely applied and well-documented probiotic genera, have been shown to regulate the progression of neurodegenerative diseases by modulating the GM (120,121). The therapeutic and preventive effects of probiotic supplementation are achieved through multiple mechanisms. For example, the two aforementioned genera can upregulate tight junction protein expression, promote mucus secretion and block the adhesion of pathogenic microorganisms, thereby reinforcing intestinal barrier function (122).

Moiseyenko *et al* (123) developed a microbial consortium of eight human commensal bacteria whose abundance is depleted in patients with PD. Their results demonstrated that this consortium alleviated motor and GI dysfunction in mice overexpressing α-Syn. Furthermore, oral administration of a single *Faecalibacterium prausnitzii* strain markedly relieved motor and GI impairments and reduced cerebral α-Syn aggregation. In addition, 16-week oral supplementation with a probiotic mixture composed of *Bifidobacterium, Lactobacillus* and *Lactococcus* species was shown to ameliorate motor functions, including gait, balance and coordination, and markedly attenuate dopaminergic neuronal loss in mice (124). Another study on combined probiotic interventions further verified that such treatment mitigates PD symptoms by reshaping microbial composition and modulating GM metabolism (125). In mouse models of PD induced by 1-methyl-4-phenyl-1,2,3,6-tetrahydropyridine (MPTP), *Lactobacillus acidophilus* alleviated PD symptoms by concurrently modulating GM composition and the GLP-1 receptor signaling pathway (126). This intervention improves motor performance, preserves neuronal viability, suppresses neuroinflammation and reinforces intestinal barrier integrity, offering a promising gut-brain axis-based strategy for neuroprotection.

A RCT enrolled 105 patients with PD-related constipation, who were randomized into the following three groups: i) Traditional Chinese medicine (Liuwei'anxiao capsules) group; ii) single-probiotic treatment group; and iii) combined therapy group. The combination therapy yielded a higher overall response rate for constipation relief compared with the other two groups. Additionally, the relative abundances of core beneficial genera, including *Bifidobacterium, Megamonas* and *Roseburia*, were restored (127). Similarly, a 12-week randomized controlled pilot study was conducted in patients with PD receiving a probiotic supplement consisting of *Bacillus licheniformis, Bifidobacterium longum, Lactobacillus acidophilus* and *Enterococcus faecalis*. The results revealed that the probiotic intervention notably reduced the UPDRS scores and alleviated symptoms of RBD in patients with PD (128). Moreover, research has found that probiotics can enhance levodopa absorption and alleviate motor and cognitive symptoms, including anxiety, depression and memory problems (129). Collectively, probiotic supplementation exerts extensive protective and beneficial effects on human health. Accumulating evidence has demonstrated that regular intake of fermented dairy products contributes to maintaining optimal mental health in daily life (130,131).

To further elucidate the clinical implications of probiotic-based therapy in PD, Danika and Supital (132) conducted the first specialized meta-analysis evaluating the motor-improving effects of probiotic interventions. Pooled analysis revealed that probiotic supplementation confers notable benefits on motor function in patients with PD vs. placebo controls, with all included studies demonstrating consistent positive therapeutic trends. Notably, probiotic treatment exhibited a favorable safety profile. Specifically, adverse events were sparse and mild, mainly manifesting as temporary GI discomfort, and no severe treatment-related complications were observed. These dual advantages of efficacy and safety provide a solid translational basis for probiotics as a long-term adjunctive management approach for PD. Despite these promising findings, multiple limitations and research gaps persist in current evidence. Considerable heterogeneity exists among available RCTs in terms of probiotic strains, dosages and intervention schedules, while small sample sizes and the absence of probiotic characteristic-based subgroup analyses further restrict generalizability. Future research should prioritize standardization of probiotic strains, dosages and experimental protocols to enhance cross-trial comparability. Additionally, integration metagenomic and metabolomic profiling to guide microbiota-based stratified intervention may promote precision probiotic medicine for PD treatment.

### Antibiotic interventions

Antibiotics are a class of chemicals used to inhibit or kill bacteria, primarily to combat infections. Antibiotics exhibit immunomodulatory, neuroprotective, anti-amyloidogenic and antioxidant properties (133–135). Their regulatory effect on microbiota is double-edged, as they can trigger dysbiosis, which is associated with the onset of various neurodegenerative diseases and psychiatric conditions, including schizophrenia and depression (136). When harnessed as a therapeutic approach, antibiotics may confer protection against neurological disorders, including neurodegenerative diseases (137,138). Next-generation sequencing and metagenomic technologies are widely used to analyze the GM in patients with PD. Research has shown that changes in GM abundance resulting from antibiotic use can directly or indirectly influence the MGBA (139,140). For instance, ceftriaxone, doxycycline derivatives and clorobiocin can directly interact with α-Syn, suppressing misfolding and aggregation (141–143). In a preclinical study, Wu *et al* (144) used antibiotic treatment to deplete the GM, reducing *Dubosiella* abundance and branched-chain amino acid levels, ultimately reversing PD progression and reducing the spread of α-Syn pathology.

Ceftriaxone, a third-generation broad-spectrum cephalosporin, exerts anti-neuroinflammatory and antioxidant activities. Its neuroprotective effects have been validated in multiple PD models established with MPTP and LPS. It suppresses ferroptosis and the activation of microglia and astrocytes, thereby alleviating neuronal damage indirectly (145–147). Similarly, minocycline (a second-generation semisynthetic tetracycline antibiotic) not only effectively crosses the BBB, but also possesses antibacterial, anti-apoptotic, anti-inflammatory and antioxidant properties. Furthermore, it can improve memory impairment by inhibiting microglia activation and neuroinflammation, thereby preserving synaptic function (148,149). Recent research indicates that minocycline nanocarriers can cross the BBB effectively, enabling PD with cognitive impairment (150). As another second-generation tetracycline antibiotic, doxycycline exhibits superior BBB permeability and a favorable clinical safety profile, and studies have shown that it ameliorates PD-related neuropathological alterations by suppressing neurodegeneration, neuroinflammation, oxidative stress and endoplasmic reticulum stress (151,152).

There is a wide variety of available antibiotics, and therefore exploring additional options with similar functions would be beneficial to mitigate the risks associated with relying on a single antibiotic. Cui *et al* (153) found that vancomycin pretreatment had multiple effects, including reducing astrocytes and microglia numbers in the SNPC, increasing fecal SCFAs levels and the abundance of *Akkermansia* and *Blautia*, improving motor symptoms and decreasing dopamine turnover. In addition, rifampin and rifaximin also have neuroprotective effects, but they were not discussed in detail (154,155).

These findings provide new insights into the effects of various antibiotics and their respective impacts on the GM of PD. However, when multiple antibiotics are present, their interactions (such as synergistic or antagonistic effects) and the cumulative burden of adverse side effects should not be overlooked. For instance, short-term treatment with a composite antibiotic disrupted GM in rats and markedly reduced SCFAs levels. While α-Syn expression in the colonic ENS remained unchanged, and it was substantially downregulated in the olfactory bulb of the forebrain (156). Accordingly, cautious use of broad-spectrum antibiotics is strongly recommended.

It is worth noting that extensive, prolonged human antibiotic use erodes drug effectiveness and exacerbates the global antimicrobial resistance crisis (157). Additionally, conventional antibiotics often fail to target pathogens selectively, indiscriminately disturbing a substantial portion of the GM and reshaping the intestinal microbial landscape. In other words, CNS effects on the GM persist even after depletion of bacteria using broad-spectrum antibiotics (158). While antibiotic exposure can be associated with neurocognitive and mood disturbances in some individuals, accumulating clinical trial evidence supports the potential benefits of antibiotics in certain neurodegenerative contexts, suggesting that they could become a promising alternative strategy against PD (159,160).

### Dietary interventions

It is hypothesized that dietary intervention, as a non-pharmacological approach, may exert a therapeutic effect on PD by influencing GM structure and host metabolism. Hegelmaier *et al* (161) conducted a 14-day dietary intervention on 16 patients with PD and collected stool samples before and after treatment. Compared with the healthy controls, the patients showed an increase in the abundance of anti-inflammatory GM following dietary intervention, and notable clinical improvements were observed. Levodopa is widely acknowledged as the gold standard for symptomatic PD treatment, but the present review does not detail its pharmacological effects. In addition, the mechanism underlying the impact of dietary protein on levodopa bioavailability has yet to be investigated thoroughly. However, it has been shown that high-protein diets can compete with levodopa for intestinal absorption and for transport across the BBB, thereby reducing its effectiveness (162–164). Furthermore, there is evidence that levodopa efficacy may be enhanced when co-administered with a low-protein diet (165). Consequently, patients on levodopa or related therapies should follow a low-protein diet. To optimize efficacy, a defined interval between protein intake and medication administration is recommended. This helps to avoid interference with absorption and metabolism, preserving efficacy and reducing adverse effects.

Cruciferous vegetables and their phytochemicals are potential neuroprotective agents. Sulforaphane, a key component of cruciferous vegetables, can improve motor symptoms and behaviors in PD by mitigating dopaminergic neuron damage and via anti-inflammatory and antioxidant actions. Multiple preclinical studies and clinical trials have demonstrated that glucosinolates possess neuroprotective effects (166–168). Another key phytochemical, indole-3-carbinol, has been verified to suppress α-Syn expression in preclinical PD rat models, thereby improving neurological function (169). Consequently, regular consumption of cruciferous vegetables (including Brussel sprouts, cabbage, cauliflower, kale and turnips) may help prevent or mitigate neurodegenerative diseases.

Owing to its abundant dietary fiber, the Mediterranean diet exerts beneficial modulatory effects on the GM (170). A 6-month RCT enrolled 72 patients with PD receiving standard therapy plus SCFAs and/or prebiotic 2′-fucosyllactose. All intervention groups showed notable motor improvements and reduced levodopa requirements, concurrent with improved intestinal barrier function (171). This finding provides some of the most direct evidence to support dietary fiber intervention as an effective strategy for optimizing clinical outcomes in PD. By contrast, Western diets, high in red meat, saturated fat and cholesterol, may exert detrimental impacts on the gut-brain axis (172). A case-control study directly linked Western dietary patterns with increased PD risk (173). Furthermore, nutritional supplements such as coenzyme Q10, lycopene and resveratrol have been shown to target pathological mechanisms, including α-Syn misfolding and neuroinflammation (174). This suggests that these supplements may have therapeutic potential and could improve the quality of life of patients.

Strategic and healthy dietary modification represents a promising interventional approach. Specifically, selective refinement of dietary ingredients exerts multifaceted regulatory effects on the pathogenesis and progression of neurodegenerative diseases. Further mechanistic investigations are essential to fill the existing research gaps and provide solid data to support the development of targeted and potent dietary intervention protocols.

### Fecal flora transplantation

FMT, also termed fecal transplantation or fecal bacteriotherapy, is a strategy with potential for reconstructing the GM. It involves transferring a liquid filtrate from processed feces of a healthy donor into the gut of the recipient to treat disease (175). This intervention aims to restore a healthy intestinal microbiota by reconstituting microbial diversity. Research has shown that FMT substantially impacts non-GI symptoms associated with neurological disorders, including constipation, in PD (176,177). Most researchers attribute these effects to mechanisms such as alterations in neurotransmitter metabolites, vagal signaling and immune activation (178).

Preclinical studies show that FMT can reduce nigral inflammation and protect dopaminergic neurons (179). For example, Sun *et al* (180) demonstrated in an animal study that FMT from healthy donors into MPTP-induced PD mice elevated striatal neurotransmitters, reduced neuroinflammation and improved motor performance. By contrast, healthy mice receiving fecal microbiota from patients with PD showed reduced striatal neurotransmitter levels and worsened motor function. Another preclinical study found that FMT from patients with PD to PD mice aggravated α-Syn overexpression and worsened α-Syn-related motor dysfunction (181). In a clinical trial, Cheng *et al* (182) conducted a RCT involving 56 patients with PD; among the 27 patients which completed the trial, no serious adverse events occurred. Compared with the placebo, FMT recipients showed notable autonomic function improvements. Collectively, this trial supported the feasibility and potential of oral FMT to aid substantia nigra restoration and accelerate GM reconstruction in PD.

Notably, findings from the foregoing preclinical animal investigations have failed to yield consistent outcomes when translated into human clinical trials. One large-scale multicenter RCT performed in Finland confirmed the favorable safety profile of FMT; nevertheless, no statistically significant intergroup differences in MDS-UPDRS Part III scores were observed between the FMT and placebo arms at the 12-month follow-up (183). Although the aforementioned study by Cheng *et al (182)* observed an improvement in the total UPDRS score, no significant difference was observed in the motor subscale. In a RCT Phase II study of 46 patients with PD conducted by Beckers *et al* (184), FMT treatment yielded a 3.1-point greater improvement in MDS-UPDRS Part III scores compared with the placebo group. The marginal improvements in objective motor scores observed in human trials are in contrast to the 40–50% enhancement in motor function post-FMT in animal models, as documented by Sampson *et al* (181). This discrepancy highlights prominent species-specific variations in the therapeutic efficacy of FMT.

A study conducted by Wang *et al* (185) demonstrated that fresh FMT alleviated exercise-associated symptoms in patients with PD, suggesting that fecal microbiota preparation protocols are key determinants of FMT therapeutic efficacy. Furthermore, the quality of the donor stool is a decisive factor in the efficacy of transplantation. Currently, only a small proportion of donated stool meets transplantation standards, and the lack of unified criteria for assessing quality restricts the widespread application of this therapy to large populations.

There are several issues regarding FMT that require attention: i) This intervention requires large sample sizes in clinical trials to establish its feasibility, and it may face challenges such as poor patient compliance and dropouts; ii) the optimal timing, dosage and frequency of transplantation remain undefined; and iii) strict adherence to ethical and regulatory standards is imperative, which is reflected in the guidance principles of the US FDA guidelines. Therefore, further clinical trials are recommended to evaluate the long-term efficacy and safety of this treatment regimen.

### VNS

The vagus nerve, the tenth cranial nerve, serves as the primary trunk of the parasympathetic nervous system. Along its descending pathway from the brain to the gut, it transmits digestive signals that regulate gastric acid secretion, GI motility and the release of digestive enzymes. In its ascending pathway from the gut to the brain, it continuously relays information, such as nutritional status, hormone levels, inflammatory signals and GM metabolites, back to the brain in real time.

Due to the extensive innervation of the vagus nerve, VNS is considered an effective treatment for various emotional disorders and clinical conditions. For example, it is currently approved by the US FDA for treating depression and epilepsy (186–189). Clinical studies have found that patients who have undergone total vagotomy of the trunk are at reduced risk of developing PD (190). Yang *et al* (191) performed unilateral intrastriatal infusion of intestinal and vagus nerve lysates from patients with PD into the striatum of rats. This intervention provokes dopaminergic neuronal degeneration and deposition across several brain regions such as the striatum and substantia nigra, initiating a prion-like pathological cascade. Injecting epigallocatechin gallate-loaded neuron-targeted nanoparticle/temperature-sensitive hydrogel into the cervical vagus nerve of mice has been shown to effectively degrade intracellular α-Syn, thereby slowing the progression of PD (192). Furthermore, Li *et al* (193) found that activation of the vagus nerve and its downstream α7nAChR-JAK2/STAT3 signaling cascade markedly mitigates inflammatory responses, implying that VNS exerts regulatory effects on neuroinflammation in PD via these molecular targets.

Extensive preclinical studies corroborate the central contribution of the vagus nerve to PD pathogenesis and highlight the therapeutic prospect of vagus-targeted interventions. However, a meta-analysis pooling six large-scale clinical trials encompassing 176 patients with PD revealed that non-invasive VNS yields small-to-moderate improvements in gait and motor function. Notably, verbal fluency, sleep-related disorders and fatigue are negatively affected while on medication (194). A separate meta-analysis comprising 156 patients with PD further demonstrated that although percutaneous VNS improved stride length, walking velocity and UPDRS-III motor ratings, effect sizes varied substantially across all outcomes (195). Similarly, a clinical study of multiple-dose transcutaneous cervical VNS also suggested that VNS has minimal effects on gait and cognition (196).

This marked discrepancy in effect sizes can be ascribed to divergent depths of mechanistic investigations between preclinical animal models and human clinical research. Preclinical animal experiments adopt standardized stimulation protocols and modeling approaches alongside invasive VNS delivery. By contrast, human trials feature pronounced variability in stimulation locations, stimulus frequencies and outcome assessment instruments, which contributes to substantial heterogeneity across treatment effect sizes. Furthermore, existing acute or subacute PD animal models fail to faithfully recapitulate the protracted 20–30-year chronic neurodegenerative progression observed in human patients; confounding pharmaceutical interventions administered to human participants may obscure or diminish the standalone therapeutic benefits of VNS.

Collectively, VNS represents a promising adjunct therapeutic strategy for PD with substantial research potential, and non-invasive VNS confers superior safety profiles relative to its invasive counterpart (197). Nevertheless, the optimal stimulation parameters, targeted symptom selection and patient stratification criteria for non-invasive VNS remain to be defined via large-scale, standardized clinical trials. In addition, exploring the mechanisms by which vagal nerve subtype heterogeneity and targeting-site variation modulate experimental outcomes will help elucidate the retrograde propagation mechanism of α-Syn and inform the rational design of disease-modifying therapies.

## 5. Summary and outlook

In summary, the global incidence of PD continues to rise annually, leading to a growing patient population that imposes substantial psychological and socioeconomic burdens on individuals and their families. Increasing research attention has been directed toward the non-motor symptoms of PD. As a core signaling hub of the gut-brain axis, GM modulates disease progression through multiple pathological mechanisms. Compositional and metabolic alterations in GM can serve as promising novel biomarkers, enabling early and accurate clinical diagnosis of PD. Emerging microbiota-modulating strategies are being developed for the prevention and treatment of PD. These strategies encompass prebiotics, probiotics, antibiotics, dietary interventions, FMT and VNS. Nevertheless, these interventions still have certain limitations and require further clinical trials to validate their efficacy and safety. The present study systematically reviewed the association between the MGBA and PD, providing innovative insights into PD pathogenesis and therapeutic development. Future research should integrate basic research with clinical translation by leveraging advanced biosensing, genomics and metabolomics technologies, in combination with diversified intervention strategies. Such integrated approaches are expected to accelerate the advancement of personalized treatment and early intervention systems for PD.

## Figures and Tables

**Table I.** GM combinations and their overall Spearman ρ correlation coefficients with corresponding blood metabolites from the published dataset of Hensen and Thiele (43).

Metabolites in the blood	Most relevant GM combination	Spearman ρ correlation coefficient
L-leucine/leucyl-leucine	*Bacteroides ovatus Roseburia intestinalis*	0.29
Butyrate	*Bacteroides uniformis Blautia wexlerae*	0.81
	*Eubacterium recatle Faecalibacterium prausnitzii*	
Nicotinic acid	*Alistipes putredinis Collinsella aerofaciens*	−0.49
	*Phocaeicola plebeius Ruthenibacterium lactatiformans*	
Pantothenate	*Bacteroides dorei Bacteroides vulgatus*	0.46
	*Faecalibacterium prausnitzii*	
Myrisitc acid	*Bacteroides uniformis Bacteroides vulgatus*	0.77
	*Faecalibacterium prausnitzii Prevotella copri*	

Each coefficient represents the overall correlation of the entire combined GM consortium, rather than individual single bacterial species. GM, gut microbiota.

## Data Availability

Not applicable.
